# The Significance of Epithelial-to-Mesenchymal Transition for Circulating Tumor Cells

**DOI:** 10.3390/ijms17081308

**Published:** 2016-08-11

**Authors:** Alexandra C. Kölbl, Udo Jeschke, Ulrich Andergassen

**Affiliations:** Department of Gynecology and Obstetrics, LMU Munich, Maistrasse 11, 80337 Munich, Germany; alexandra.koelbl@med.uni-muenchen.de (A.C.K.); ulrich.andergassen@med.uni-muenchen.de (U.A.)

**Keywords:** circulating tumor cells, epithelial to mesenchymal transition, marker, prognosis, cancer, metastasis

## Abstract

Epithelial to mesenchymal transition (EMT) is a process involved in embryonic development, but it also plays a role in remote metastasis formation in tumor diseases. During this process cells lose their epithelial features and adopt characteristics of mesenchymal cells. Thereby single tumor cells, which dissolve from the primary tumor, are enabled to invade the blood vessels and travel throughout the body as so called “circulating tumor cells” (CTCs). After leaving the blood stream the reverse process of EMT, the mesenchymal to epithelial transition (MET) helps the cells to seed in different tissues, thereby generating the bud of metastasis formation. As metastasis is the main reason for tumor-associated death, CTCs and the EMT process are in the focus of research in recent years. This review summarizes what was already found out about the molecular mechanisms driving EMT, the consequences of EMT for tumor cell detection, and suitable markers for the detection of CTCs which underwent EMT. The research work done in this field could open new roads towards combating cancer.

## 1. Introduction

**Figure 1:**
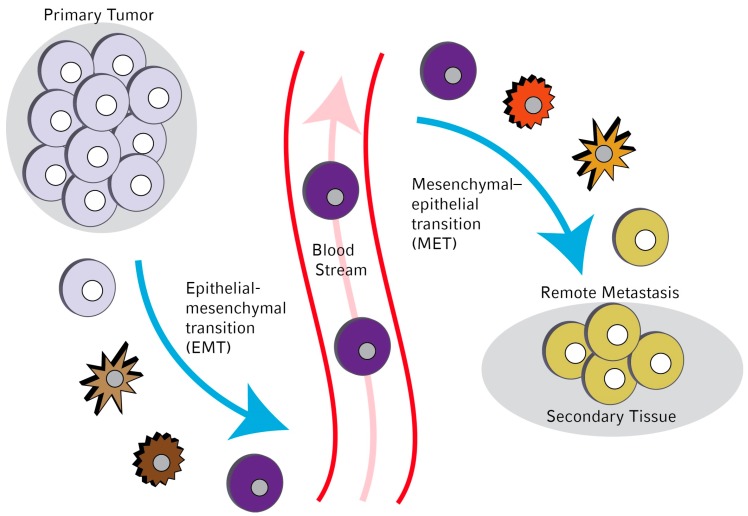
Remote metastasis formation by circulating tumor cells (CTCs) undergoing epithelial to mesenchymal transition-mesenchymal-to-epithelial transition (EMT-MET) changes of their cellular characteristics. Single cells dissolve from the primary tumour, adopt mesenchymal properties, enabling them to invade into the blood stream, after extravasation CTCs regain epithelial characteristics, thus they can seed in secondary tissues, building bud for remote metastasis formation.

Frequently, during the process of tumor outgrowth, single cells dissolve from the primary tumor and enter circulation. These cells are called “circulating tumor cells” (CTCs) [1]. If they extravasate again they can settle down in different sites of the body and are considered to be the main reason for remote metastasis formation. They can also enter bone marrow and stay there for long time periods in a certain state of dormancy [2,3,4,5,6,7]. These cells are called “disseminated tumor cells” (DTCs). Both, CTCs and DTCs have a prognostic relevance for affected patients [1,8,9] and their presence/absence is already included in the international tumor staging systems [10,11,12]. As DTCs could only be obtained by bone marrow aspirations, CTCs are in the focus of cancer research. But also the detection of CTCs bears some obstacles: first, their number, in comparison to the surrounding blood cells is rather small (1/10^6^ blood cells [13]), so that most of the available detection methods require an enrichment step [14]. Second, during the process of detachment from the primary tumor and invasion of the blood stream these cells undergo a number of phenotypical changes. CTCs which are in fact of epithelial origin change their properties, like cell adhesion, cell mobility, and invasiveness, and loss of epithelial markers, so they become mesenchymal-like cells in a process called epithelial-to-mesenchymal transition (EMT) [15,16]. A graphical summary of the EMT process is presented in Figure 1. Thereof result difficulties for detection, because it gets more difficult to distinguish these cells from mesenchymal blood cells [17,18], and tumor cells can no longer be related to their primary tumor. This process is reversed, when these cells extravasate again and settle down in distant tissues of the body to form a metastatic bud (mesenchymal-to-epithelial transition (MET) [19,20,21]). For the reason of this heterogeneity of CTCs lots of research work on CTCs undergoing EMT had been done in the last five years and the results are summarized in the following.

## 2. Historical Background

One of the rather early findings in CTC-EMT research was, that CTCs show EMT and stem cell characteristics, and it was assumed, that this might be an indicator for therapy resistance and an inferior prognosis [22], as CTCs displaying the mesenchymal phenotype are believed to have an increased metastatic potential [23]. Additionally, this fact creates the need to adjust detection methods [18,24]. Another suggestion was that tumor cells do not migrate throughout the body as single cells but as clusters, so called tumor cell microemboli (TCM), creating a protective surrounding for the dissolved tumor cells, especially as apoptotic marker are absent from these cell clusters [21,25,26]. The first report on EMT markers in CTCs was published by Kallergi et al. [27] in 2011. They analyzed the expression of two EMT markers, Twist and vimentin, in CTCs of breast cancer patients by immunofluorescence staining. CTCs expressing these two markers were found with higher frequency in metastatic breast cancer patients than in patients with early stages of the disease, pointing towards the malignant potential of EMT [27]. Furthermore, CTCs coexpressing epithelial, EMT and cancer stem cell (CSC) markers were found in patients with metastatic diseases [28]. Bonnomet et al created a dynamic model in 2012, examining the EMT process and CTC formation in a mouse xenograft model. They found that EMT already occurs within the primary tumor, conceding to cells the possibility to intravasate and form CTCs. The inverse process, mesenchymal-to-epithelial transition (MET) is proposed to occur in secondary organs, giving rise to metastatic outgrowth [29,30]. The analysis of CTCs for EMT and stem cell markers in primary breast cancer patients could in fact not be correlated with the prognostic clinical markers but again the need for an adjustment of detection systems and as well as for the examination of their prognostic relevance is highlighted [31]. To overcome the obstacle of CTC isolation, a negative depletion enrichment methodology, removing CD45^+^ cells first, was proposed [32]. In another study the presence of EMT-CTCs in Her2^+^ metastatic breast cancer patients was shown by an assessment of EMT-inducing transcription factors. In almost 90% of the CTCs at least one EMT-associated transcription factor was upregulated, pointing towards the presence of a high number of EMT-CTCs [33]. Subsequently, it was demonstrated that EMT-CTCs in metastatic breast cancer patients under high dose chemotherapy and autologous hematopoietic stem cell transplantation, are a prognostic factor for shorter progression free survival (PFS) and relapse [34]. In patients with hepatocellular carcinoma especially vimentin and Twist expression in CTCs could serve as a biomarker for the evaluation of metastasis formation and prognosis of the disease [35]. Charpentier et al. proposed a model of metastasis formation in breast cancer, claiming that CTCs with both, EMT and CSC characteristics were necessary for metastatic outgrowth. CSCs and EMT-CTCs have a flexible cytoskeleton and anoikis resistance, so they can survive in the blood stream, and additionally the expression of vimentin leads to a formation of microtentacles, which help the cells to reattach to new tissue sites. The authors therefore conclude that the combination of these characteristics increases metastatic efficiency [36]. These findings were confirmed in a later study, finding that CTCs expressing ALDH1A1 together with nuclear expression of twist were more frequently detected in patients with metastatic breast cancer [37]. As normal CTCs and EMT-CTCs vary in size so that in an enrichment process, only by size many cells would escape isolation, an interesting approach was published by Ito et al.: they used a telomerase–specific, replication selective oncolytic adenoviral agent tagged to a GFP (green fluorescent protein) protein. Blood from patients with gastritic cancer was infected with this agent and green fluorescent cells could be detected easily and were recognized as a prognostic marker for overall survival (OAS) [38]. EMT-characteristic markers like E-cadherin, vimentin, N-cadherin, Twist, and SNAI1/2 were also examined in tissue of lymph node metastases. Interestingly, and also in contrast to the primary tumor, E-cadherin expression is high, EMT-transcription factors are upregulated, while Ki67-rates are decreased in lymph nodes, which might be kind of a survival strategy [39]. A recent approach describes the classification of CTCs by the measurement of expression of EMT markers by RNA-ISH (RNA in situ hybridization). Three types of CTCs could be discerned: epithelial, epithelial/mesenchymal, and mesenchymal CTCs. This classification is rather important as mesenchymal CTCs are frequently found in patients with metastatic stages of the disease, giving hints for prognosis and treatment [40,41,42]. EMT and stem cell specific transcripts were also shown to correlate with clinical stage and can be detected in patients negative for epithelial marker expression [43]. Thereof arises the necessity to identify different tumor cell subpopulations, which might be able by different methods, but also a standardization of these variegated techniques is important [44]. Furthermore, it has to be taken into account, that certain treatment strategies might alter phenotypes of CTCs, so that they might be able to escape detection routines, as was shown for colorectal cancer patients, who were treated with bevacizumab for longer time periods [45].

## 3. Molecular Mechanisms Leading to EMT in Circulating Tumor Cells (CTCs)

To clarify the mechanisms leading to EMT in CTCs and the analysis of underlying signal transduction cascades would make up a step stone for combating metastasis formation. One of the earliest findings on this topic was that inflammation seems to increase the EMT-rate in pancreatic cancer [26,46]. Acidosis seems to be another factor leading to EMT [47,48]. Also hypoxia was found to play a role in early events of EMT, as described in a study on multiple myeloma. Hypoxia decreased E-cadherin expression, resulting in less cell adhesion and entry of the dissolved cells into the circulation [49], but also the whole tumor microenvironment is able to stimulate cell migration and invasion. The tumor stroma can for example, initiate EMT on the invasive front of the tumor, which also gives signals back to the primary lesion [50]. Signal-peptide-CUB-EGF-like domain containing protein3 (SCUB3) is an example of an important signaling molecule, as it promotes tumor angiogenesis and EMT. A knockdown of this gene results in lower vascular permeability and decreased metastatic potential in non-small cell lung cancer NSCLC, and could thereby be a potential therapeutic target [51]. The role of fibroblast growth factor receptors 1 and 3 (FGFR1/3) was described in bladder cancer. FGFR1 is a transcription factor for the expression of mesenchymal genes like *ZEB1* and *vimentin*, FGFR3 in contrast influences the expression of epithelial markers like E-cadherin and p63. The interplay between these two receptors seems to play a role in the outgrowth of bladder cancer [52]. In prostate cancer Hsp27, a molecular chaperone, drives EMT via IL-6-mediated modulation of STAT/Twist. Hsp27 inhibition leads to a decreased number of CTCs, so it might become rather interesting for therapeutic strategies in prostate cancer [53]. Hepatocyte growth factor and its receptor c-Met are associated with tumor progression and metastasis in hepatocellular carcinoma. In the CTCs of this cancer entity, a high expression of these molecules comes along with an EMT phenotype, due to a lack of CpG-methylation in the c-Met region [54]. Forkhead box protein M1 (FOXM1) in contrast was a key regulator of EMT in breast cancer, as it binds and stimulates the promotor of Slug, which is responsible for EMT-promotion. Via this signaling pathway FOXM1 leads to metastasis formation [55]. In colorectal cancer EMT is induced by PLS3 via TGFβ-signaling cascades, resulting in invasive properties of cancer cells [56], but also special tumor treatments were shown to promote metastasis: in hepatocellular cancer, transcatheter arterial embolization is a common palliative treatment, but it was shown that it simultaneously upregulates hypoxia-inducible factor 1a (HIF1a) and epithelial to mesenchymal marker proteins like N-cadherin and vimentin thereby stimulating the metastatic potential of tumor cells [57]. Also, core needle biopsy in breast cancer seems to increase EMT and facilitates additionally the release of CTCs, which might contribute to remote metastasis formation [58]. Some tumor treatments are also known to increase the number of TCMs, and these clusters are more resistant to apoptosis as single tumor cells, giving rise to metastasis with a higher probability [26]. However, there are already also treatments which abolish EMT, like gemcitabine treatment of NSCLC-patients. It not only decreases the number of EpCAM (epithelial cell adhesion molecule) positive CTCs, but also inhibits EMT via the HGF/c-Met pathway [59]. Another signaling cascade regulating EMT was presented by Yuan et al. They could show, that an inhibition of p-Akt led to an upregulation of miR-200s, which in turn leads to a downregulation of EMT markers [60]. In bladder cancer, miR-34a has a suppressive role for angiogenesis and metastasis by regulating EMT-related proteins [61]. Another study demonstrates, that the knockdown of multiple kinases, like MAPK7 (mitogen-activated protein kinase 7), induces the expression of epithelial markers, inhibits cell migration, and maintains epithelial phenotypes, thereby reducing tumor invasiveness [62]. Also Leucine Zipper Transcription Factor-like1 (LZTFL1) seems to convey protective effects to lung epithelial cells by regulation of EMT-associated genes. LZTFL1 maintains an epithelial phenotype and inhibits mechanisms leading to EMT [63]. EMT furthermore induces tissue factor (TF), which in turn stimulates coagulation leading to EMT-positive CTCs or CTC-clusters, which have a great metastasizing potential. Silencing of ZEB1 inhibits TF-expression while Snail stimulates its expression. These EMT-TF-axis creates a new target for therapeutic interventions in the process of metastasis formation [64].

## 4. New CTC-Detection Strategies

By the fact, that even CTC-negative patients could develop remote metastasis due to an escape of EMT-CTCs to CTC detection, it became clear, that new techniques for the detection of such CTCs had to be developed [17,65]. CTCs, which undergo EMT sometimes present with an “intermediate” phenotype. Such cells were detected five years ago in patients with metastatic non-small cell lung cancer (NSCLC) by a fluorescent co-staining of vimentin and cytokeratins [66]. At the same time, it was discovered that epithelial tumors are characterized by a complex aneuploidy, which is inherited by the dissolved, circulating tumor cells, which therefore do not express CD45 or cytokeratins, but can be detected based on this chromosome rearrangement [67]. Another approach is to use CD146 to detect EpCAM-negative tumor cells and CD49f for the detection of CK-negative cells, improving detection rates [68]. The CTCscope method, which was published in 2012, is based on an RNA-ISH detecting epithelial as well as EMT-markers from blood samples. The advantage of the method is a simultaneous enumeration and characterization only of viable cells [69]. Another interesting approach is to sort cells by size is the DC impedance-based microcytometer. Thereby it could be discriminated between blood and tumor cells without the need of cell labelling [70]. In 2013 an approach for the detection of CTCs in NSCLC by the use of CK-coated beads, and they found a good correlation of tumor cell counts with the clinical history of malignancy of the respective tumors [71]. A multicolor detection system, which was based on flow cytometry was presented by Watanabe in 2013. After a CD45-depletion cells were fixed and labelled with fluorescently labelled antibodies against CD45, EpCAM, and CK, helping to distinguish different levels of EpCAM expression. Additional antibodies against EMT-markers could also be used in this system, allowing a characterization of the tumor cells [72]. A high throughput system for EMT-CTC detection could also be a microchip filter device, which sorts cells for Caveolin-1, a marker, which was shown to be upregulated during EMT-process [73]. Another interesting marker gene for CTC-isolation and enumeration purposes could be cell-surface vimentin (CSV), which is only expressed on cancer cells and never found on the surface of healthy blood cells. With the help of monoclonal antibody 84-1 CSV-positive cells can be filtered with high specificity, and CTC-counts could be related to therapeutic response [74]. A recently published technique used miRNA in situ hybridization for tumor cell detection. miRNA-21, a known onco-miRNA, has been shown to be a good target for this method, as it is only expressed in tumor cells, in which EMT takes place [75]. Furthermore, a rather complete depletion of white blood cells can be achieved by the introduction of a single-step treatment of the cells with sulfuric acid, creating a certain nanoscale roughness, which results in an increased binding of CD45-positive white blood cells to CD45-conjugated surfaces. Thereby isolation and characterization of tumor cells is increased [76]. A novel EpCAM independent enrichment strategy was proposed by Schneck et al. in 2015: they combined different antibodies specific for cell surface proteins and components of the extracellular matrix (ECM) components. The capture molecules (Trop2, CD49f, c-Met, CK8, CK44, ADAM8, CD146, TEM8, CD47) were first tested in single- and multi-spot arrays with breast cancer cell line with known EpCAM-expression patterns. EpCAM-low/negative cells could be captured easily by this method, so that patient samples were used for further experiments. EpCAM-negative CTCs could be isolated thereby and the malignant nature of those cells could be shown by a comparative genomic hybridization, demonstrating again the importance for detection of EpCAM-negative tumor cells [77]. The CanPatrol CTC enrichment technique first isolates CTCs via filter-based method, then CTCs were classified according to their EMT-markers by RNA in situ hybridization. It was recognized, that the number of epithelial CTCs was related to tumor size, the cells with mixed EMT/epithelial properties correlated to tumor number and mesenchymal CTCs could be related to metastasis formation, highlighting the usefulness of the presented methodology [78]. The most recent approach for EMT-CTC detection comes from Pramanik et al. who describe the use of multifunctional multicolor nanoprobe assay, which is used for the capturing and mapping of heterogenous CTCs [79].

## 5. Markers for Epithelial to Mesenchymal Transition-Mesenchymal-to-Epithelial Transition (EMT-CTC) Detection

A rather important topic in the detection of CTCs which underwent EMT is of course, to find appropriate detection markers or marker panels. In a microfluidics-based PCR system expression profiles from 84 EMT-related genes were analyzed in blood samples of prostate cancer patients. Although gene expressions were quite heterogenous, some marker genes common for mesenchymal cancer cells could be identified: IGF1, IGF2, EGFR, FOXP3, and TGFB3. Furthermore, some EMT-related genes were found to be expressed commonly: PTPRN2, ALDH1, ESR, and WNT5A. Therefore it could be concluded that the analysis of the expression of EMT-markers provides opportunities for a personalized treatment of some cancer entities [80]. In colorectal cancer, Plastin3 (PLS3) was identified as a marker for EMT-CTCs, helping to detect CTCs as this marker is not expressed in healthy blood cells. It was furthermore recognized to be a marker for metastatic CTCs, conveying a prognostic relevance [81]. In early breast cancer, a high expression of MMP1 could be detected in EMT-CTCs, which could additionally be correlated to tumor grade [82]. Furthermore, EGFR seems to play an important role in EMT process. An activation of EGFR signaling in MCF7 cells led to an increase of EMT-phenotypes, inhibited apoptotic events, and induced the loss of cytokeratin expression so that the analysis of EGFR could be an important prognostic and predictive marker in breast cancer [83]. An analysis of CTCs from blood samples of high risk endometrial adenocarcinoma patients (grade 3, stage IB–IV) showed a high plasticity in the expression of EMT markers like ETV5, NOTCH1, SNAI1, TGFB1, ZEB1, and ZEB2. Furthermore, markers of stemness and potential therapeutic targets could be found within this analysis, demonstrating the heterogeneity of CTCs [84,85,86]. The distinct expression of cytokeratin, N-cadherin, and CD133 was examined in metastatic breast cancer samples. Also, for this small marker panel, a strong heterogeneity could be demonstrated, outlining the importance of tumor cell characterization [87]. Doublecortin-like kinase1 (DCLK1) is regarded to be a stem cell marker in pancreas carcinoma and is additionally upregulated in other tumor entities. It also is known to be a regulator of EMT and is therefore involved in metastasis formation. The measurement of DCLK1 levels in serum samples and of its expression in CTCs could hence be an important marker for tumor malignancy [88] and EMT. It was shown that it might prevent the hepatocellular carcinoma growth by an miRNA dependent mechanism [89]. The correlation of two famous markers, CK and vimentin, in breast cancer and their significance for patient outcome was described by Polioudaki et al. They used blood samples from metastatic breast cancer patients and breast cancer cell lines to calculate the ratio of CK/vimentin, but especially in the patient samples CK/vimentin ratios varied a lot, displaying again the heterogeneity of CTCs undergoing EMT [90]. For squamous cell lung cancer (SQCLC) fibroblast growth factor 1 was used for FISH and immunocytochemical detection of CTCs which had undergone EMT [91]. After the isolation of CTCs from blood of patients with pancreatic ductal adenocarcinoma (PDAC) via size-based filtration device and an immunofluorescence staining of the isolated cells for the EMT-marker ZEB1 and the epithelial marker CK, the cells were analyzed for KRAS (proto-onkogene) mutations. It was shown, that patients bearing a KRAS mutation had a significantly better survival rate, attributing KRAS marker properties [92]. Another study demonstrated the presence of EMT markers like BMI1 and TWIST1 and stem cell markers like CD133 and ALDH1A1 in CTCs, concluding that cells carrying stem cell features are present as well in the primary tumor as in CTCs [93]. Vimentin and Ki67 were furthermore tested for their prognostical value in CTCs of patients with advanced prostate cancer. Both are well-characterized proliferation and EMT markers. If one or both markers were found to be overexpressed in patient samples, the respective patients had poorer survival outcomes [94]. Another approach to characterize CTCs from breast cancer patients was done by Hensler et al. They performed a gene expression profiling with a panel of 55 breast cancer associated genes on enriched CTCs and peripheral blood mononuclear cells from breast cancer patients. The genes, which were found to be overexpressed in the CTC samples were associated with functions involved in the proteolytic degradation of the ECM and in the EMT-process [95]. A characterization of single CTCs from ovarian cancer was done via multiplex PCR, in order to identify therapy resistant tumor cells. The multimarker gene panel consisted of genes for epithelial (EpCAM, Muc-1, CK5/7), EMT (N-cadherin, vimentin, Snai1/2, CD117, CD146, CD49f) and stem cell (CD44, ALDH1A1, Nanog, Sox2, Notch1/4, Oct4, Lin28) features. Single cells were isolated by micromanipulation and most of them were found positive for stem cell and EMT markers, but expression was quite heterogenous, making further analysis indispensable [96]. A similar analysis using multimarker qPCR was also done for metastatic breast cancer. From this study it was also concluded that CTCs are quite heterogenous and analysis has to be done for each single patient to be able to treat the patients accordingly [97].

## 6. Conclusions

Epithelial to mesenchymal transition plays a major role in tumor formation and metastatic outgrowth and has been in the focus of research in the last few years. It has extensive consequences for the detection of circulating tumor cells, which was already introduced as a prognostic parameter in the international tumor staging systems. However, as most of the systems for the detection of circulating tumor cells were based on the epithelial cell surface marker EpCAM, which is downregulated within the EMT process. Therefore new ways for the detection and also for the enrichment of CTCs have to be explored, otherwise a part of the circulating tumor cell population escapes detection. EMT-associated genes and proteins have to be included in the tumor cell detection systems to ensure diagnosis and to reinforce the predictive value of CTCs. As CTCs contribute to a large extent to metastasis formation, it is rather important to characterize and treat them as accurately as possible, to increase the therapeutic efficiency and to reduce side effects. Exploring EMT-processes also conveys chances for tumor therapy. EMT and the signal transduction pathways within this process might serve as potent targets for therapeutic approaches. Inhibiting these signal transduction cascades by specifically tailored drugs could help to diminish or even abolish metastasis formation, maybe even without doing harm to healthy cells. Analyzing and understanding the mechanisms, which lead to EMT, especially in CTCs, which are the main root of remote metastasis formation, could therefore give rise to new treatment strategies. However, we are still at the beginning of exploring the complex underlying mechanisms which lead from tumor cell dissociation from the primary tumor through EMT towards the formation of metastasis, including MET. Lots of work still has to be done in this field, but it might offer promising perspectives for the future.
